# Non-surgical management of knee osteoarthritis: a stepped conservative care paradigm for early-stage disease and high-risk populations

**DOI:** 10.3389/fsurg.2026.1941764

**Published:** 2026-09-22

**Authors:** Yongwei Shang, Zhenfeng Tian

**Affiliations:** Department of Orthopedic Surgery, Shijiazhuang People’s Hospital, Shijiazhuang, China

**Keywords:** combined modality therapy, conservative treatment, exercise therapy, osteoarthritis, knee, weight reduction programs

## Abstract

Knee osteoarthritis (KOA) is a leading cause of chronic pain and physical disability in the aging global population, placing an immense burden on healthcare systems. Despite its rising incidence, clinical practice and research remain disproportionately focused on late-stage joint replacement, neglecting early-stage preventive and conservative care for high-risk individuals. To address this gap, this narrative review searched major databases including PubMed/MEDLINE, Embase, Web of Science, and the Cochrane Library for clinical guidelines, high-quality systematic reviews, and randomized controlled trials focusing on non-surgical interventions for early KOA and high-risk populations. Search terms included “early knee osteoarthritis,” “non-surgical treatment,” “exercise therapy,” and “weight management”. Literature analysis demonstrates that a structured, “lifelong, stepped” non-surgical paradigm yields significant clinical efficacy. Specifically, combining dietary weight loss with physical exercise optimally reduces mechanical joint loading and systemic inflammatory markers; targeted muscle strengthening, alongside mind-body exercises like Tai Chi or yoga, improves the joint microenvironment and neuromuscular control; biomechanical orthoses and physical therapies offer substantial short-term pain relief; and topical or systemic NSAIDs combined with intra-articular injections effectively manage refractory acute symptoms. Crucially, continuous clinical supervision and structured patient education are vital for enhancing long-term treatment adherence and delaying radiological disease progression. Ultimately, implementing early, proactive non-surgical interventions, grounded in patient education and integrating weight management, targeted exercise, and multimodal stepped therapies, effectively alleviates pain, restores physical function, and delays or entirely prevents the need for future joint replacement.

## Introduction

The knee joint, structured by the confluence of the patella, femoral condyles, and tibial plateau, is uniquely vulnerable to degenerative pathologies owing to its sophisticated anatomy and substantial weight-bearing responsibilities (1). Clinically, knee osteoarthritis (KOA) manifests as a multifaceted mechanobiological disorder. It is pathologically characterized by the progressive degradation of articular cartilage, aberrant subchondral bone remodeling, and chronic synovitis, culminating in a debilitating cycle of unremitting pain and functional impairment (2). While the etiology of KOA is multifactorial—encompassing advanced age, hereditary susceptibility, previous joint trauma, and metabolic dysregulation—elevated body mass index (BMI) and obesity represent the most pivotal modifiable risk factors (3). Consecutively, this unrelenting structural deterioration not only compromises the patient's independent mobility but also amplifies the risk of systemic sequelae, notably cardiovascular disease, thereby establishing KOA as a leading catalyst of global disability within aging populations (4).

Musculoskeletal disorders impose an immense societal and financial strain on global healthcare systems, a burden disproportionately spearheaded by KOA, which encapsulates approximately 60.8% of all osteoarthritis-induced disability (5). Epidemiological trajectories indicate that the worldwide prevalence of osteoarthritis effectively doubled between 1,990 and 2019. By 2020, KOA afflicted an estimated 365 million adults globally, with current projections forecasting a further 74.9% expansion by 2050 (1, 5). Driven relentlessly by demographic shifts toward an aging populace and the concurrent obesity pandemic, this escalating trend inflicts severe socioeconomic repercussions. These ramifications span from compromised workplace productivity and diminished quality-adjusted life-years (QALYS) to skyrocketing direct medical expenditures (6, 7). Consequently, mitigating this public health crisis necessitates a robust intensification of translational and clinical research dedicated to optimizing therapeutic interventions, a paradigm shift essential for alleviating both individual morbidity and the compounding economic strain on healthcare infrastructures.

While knee osteoarthritis (KOA) imposes a substantial global epidemiological burden, contemporary orthopedic research and clinical practice remain disproportionately concentrated on late-stage surgical interventions, most notably total knee arthroplasty (TKA), at the expense of comprehensive early-to-mid-stage therapeutic management (8). Current literature extensively addresses novel arthroscopic techniques, advanced pharmacotherapies, and molecular pathogenesis. Concurrently, within the surgical community, there is a discernible paradigm shift from joint replacement toward joint-preserving surgeries (9, 10). Despite these advancements, a significant clinical gap persists regarding secondary prevention and lifestyle interventions for individuals exhibiting subclinical presentations, mild symptoms, or radiographically minor joint degeneration. Although current clinical discourse heavily gravitates toward refining operative outcomes, less emphasis is placed on proactive strategies to stall disease progression in younger, active patients diagnosed with early-stage KOA. Implementing structured non-surgical interventions at these preliminary stages is critical to delaying or entirely avoiding the long-term necessity for joint arthroplasty, thereby mitigating the lifetime trajectory of severe joint disability.

Therefore, addressing the clinical needs of the vast cohort of individuals with high-risk predispositions or early-stage KOA—specifically by preventing disease deceleration and averting future surgical requisites—constitutes a paramount objective in contemporary public health. To address this imperative, we propose a novel conceptual framework centered on a lifetime, stepped non-operative therapeutic paradigm, specifically designed to proactively arrest the pathophysiological cascade of early-stage KOA. Specifically, this review aims to: (1) synthesize contemporary evidence on risk identification and diagnostic metrics for early KOA, (2) evaluate the clinical efficacy and evidence certainty of multi-modal conservative therapies (lifestyle, exercise, biomechanical, physical, and pharmacological). (3) propose a phenotype-targeted, stepped non-surgical clinical pathway. And (4) highlight the indispensable role of clinician supervision and patient education in delaying structural disease progression. Conclusively, this review underscores the foundational axiom that secondary and tertiary prevention strategies yield far greater clinical and socioeconomic efficacy than reactive therapeutic modalities.

## Methods

To establish a rigorous and clinically relevant evidence base for this narrative review on stepped conservative KOA management, literature selection was governed by a strict screening protocol. A comprehensive search was performed across PubMed/MEDLINE, Embase, Web of Science, and the Cochrane Library to identify peer-reviewed studies published between January 1, 2021, and December 31, 2025. This search window was defined to ensure the integration of contemporary high-level evidence in non-surgical therapeutics. Search strategies utilized Medical Subject Headings (MeSH) and free-text terms, including: “knee osteoarthritis”, “conservative management”, “stepped care”, “early screening”, “exercise therapy”, “weight management”, “biomechanical therapy”, “physical therapy”, “pharmacological management”, and “digital health”. To minimize bias and ensure methodological quality, eligibility was restricted to prospective randomized controlled trials (RCTs), systematic reviews, meta-analyses, and evidence-based clinical practice guidelines. Conversely, low-quality trials characterized by small sample sizes, high risk of bias, or an exclusive focus on end-stage disease (e.g., Kellgren-Lawrence grade IV) and surgical interventions (e.g., arthroplasty) were excluded. Retrieved data were qualitatively synthesized to construct a structured, non-operative therapeutic pathway optimized for early-to-mid-stage KOA.

## Pathogenesis of knee osteoarthritis

Knee osteoarthritis (KOA) is no longer classified as a passive, wear-induced degenerative pathology, but rather as an active, multifactorial disruption of joint mechanobiology and cellular homeostasis (11). Its onset is driven by a complex interplay of systemic and local variables, including advanced age, genetic predisposition, elevated body mass index (BMI), acute trauma, and joint biomechanical overload (12). Under physiological conditions, joint longevity relies on a precise metabolic equilibrium within the avascular articular cartilage, maintained cooperatively by resident chondrocytes and the surrounding extracellular matrix (ECM) (13). In the osteoarthritic microenvironment, this homeostasis is disrupted by an influx of pro-inflammatory cytokines IL-1β, TNF-α and IL-6, derived from activated macrophages and synoviocytes (14, 15). These biochemical mediators initiate a robust catabolic cascade, upregulating MMPs that systematically cleave essential ECM components, including type II collagen and aggrecan (16, 17). Ultimately, this chronic inflammatory state and concurrent oxidative stress converge on the PI3 K/AKT signaling pathway, accelerating chondrocyte apoptosis and establishing a self-perpetuating cycle of tissue degradation (14, 18).

Beyond persistent inflammatory and catabolic cascades, KOA progression involves a profound failure in cellular quality control mechanisms and epigenetic reprogramming. Chondrocyte autophagy exhibits a distinct, phase-specific character during the disease trajectory: while initially upregulated as an adaptive survival mechanism against mechanical and metabolic stressors, progressive or dysfunctional autophagy in late-stage KOA often converges with apoptotic pathways, accelerating articular cartilage erosion (19, 20). This cellular recycling pathway is tightly controlled by the mammalian target of rapamycin (mTOR) axis, which integrates signals from upstream PI3 K/AKT and AMPK cascades; consequently, aberrant mTOR expression compromises downstream cartilage structural homeostasis (20, 21). Operating alongside these intracellular pathways, distinct epigenetic networks—including DNA methylation, specific histone modifications (such as HDAC-mediated MMP-13 upregulation), and non-coding RNA circuitry (lncRNAs, miRNAs, and circRNAs)—critically dictate joint susceptibility and drive catabolic phenotypic shifts without altering the primary DNA sequence (22, 23). Ultimately, the net rate of joint degeneration is determined by a baseline metabolic imbalance: while catabolic enzymes and inflammatory signals drive tissue destruction, endogenous anabolic factors—such as TGF-*β*, IGF-1, and lubricin—serve as pivotal counter-regulatory mechanisms that suppress oxidative stress and downregulate catabolic gene transcription (24).

## Risk factors and diagnostic criteria of KOA

To systematically map and quantify lifetime risk factors for incident knee osteoarthritis (KOA), evidence from a comprehensive meta-analysis comprising 131 studies and over 150 variables has provided pivotal diagnostic clarity (25). According to this high-certainty evidence, prior knee injury, advanced age, and elevated bone mineral density (BMD) are robustly associated with an increased incidence of radiographic KOA. Crucially, from a preventative standpoint, the analysis confirmed with moderate-to-high quality evidence that modifiable lifestyle and mechanical factors, specifically overweight/obesity, previous joint trauma, and intensive occupational physical activity, serve as independent catalysts for incident disease. These specific variables should therefore constitute the core actionable targets for secondary preventive frameworks. Interestingly, while crude epidemiological trends often highlight a higher crude prevalence in females, rigorous adjusted analyses within this pooled evidence indicated that biological sex alone, when isolated from age-related endocrine shifts, exhibits a negligible direct effect on the baseline risk of developing incident KOA (26).

The clinical diagnosis of knee osteoarthritis (KOA) is systematically established through the synthesis of patient-reported constitutional symptoms and objective physical examination findings. Diagnostically, the clinical presentation is characterized by unremitting localized pain, morning or activity-induced joint stiffness, and progressive functional limitations. These subjective hallmarks are validated during physical assessment by characteristic phenotypic signs, including palpable or audible crepitus, periarticular bony enlargement, joint line tenderness, gross deformity, and a restricted range of motion. A definitive clinical and radiographic diagnosis of KOA is typically validated according to the American College of Rheumatology (ACR) criteria. This requires the manifestation of recurrent knee pain within the preceding month, concurrently accompanied by at least two distinct clinical or imaging hallmarks. These diagnostic benchmarks include an age threshold of 50 years or older, prolonged morning stiffness persisting for a minimum of 30 minutes, and the detection of palpable or audible crepitus during active joint mobilization. Radiographically, the diagnosis is confirmed via standing or weight-bearing films that demonstrate hallmark degenerative pathologies, specifically asymmetric joint space narrowing, subchondral bone sclerosis, subchondral cyst formation, or marginal osteophyte development (27).

Given the frequent discrepancy between clinical symptom severity and objective joint destruction, evaluating structural progression requires standardized staging across multiple modalities. This morphological assessment is routinely achieved through conventional weight-bearing radiography, which remains the diagnostic gold standard for structural staging, supplemented by magnetic resonance imaging (MRI) for multi-planar soft tissue profiling and localized arthroscopic visualization for the direct, real-time assessment of early chondral lesions (12). Among these diagnostic modalities，the radiographic Kellgren-Lawrence (K-L) classification system (Table 1) scales as the global gold standard, maintaining its position as the most universally implemented framework for stratifying the structural severity of joint degeneration (Figure 1). Despite the emergence of advanced cross-sectional imaging, the enduring clinical dominance of the K-L grading matrix stems from its exceptional cost-effectiveness, widespread reproducibility, and established correlation with long-term prognostic outcomes in epidemiological research (28).

**Table 1:** Radiographic classification and structural severity scoring of knee osteoarthritis according to the Kellgren-Lawrence (K-L) grading system.

K-L Grade	Severity	Joint space narrowing	Osteophyte formation	Subchondral sclerosis
Grade 0	None	Normal joint space width	None	No degenerative alterations
Grade 1	Doubtful	Doubtful narrowing of the joint space	Possible osteophytic lipping	None
Grade 2	Minimal	Suspected or possible narrowing	Definite osteophyte formation	No bone deformity
Grade 3	Moderate	Unambiguous (definite) narrowing	Multiple moderate osteophytes	Initial subchondral sclerosis; potential contour deformity
Grade 4	Severe	Marked or near-total joint space loss	Large, prominent osteophytes	Intense subchondral sclerosis; pronounced deformity of bone ends

**Figure 1:**
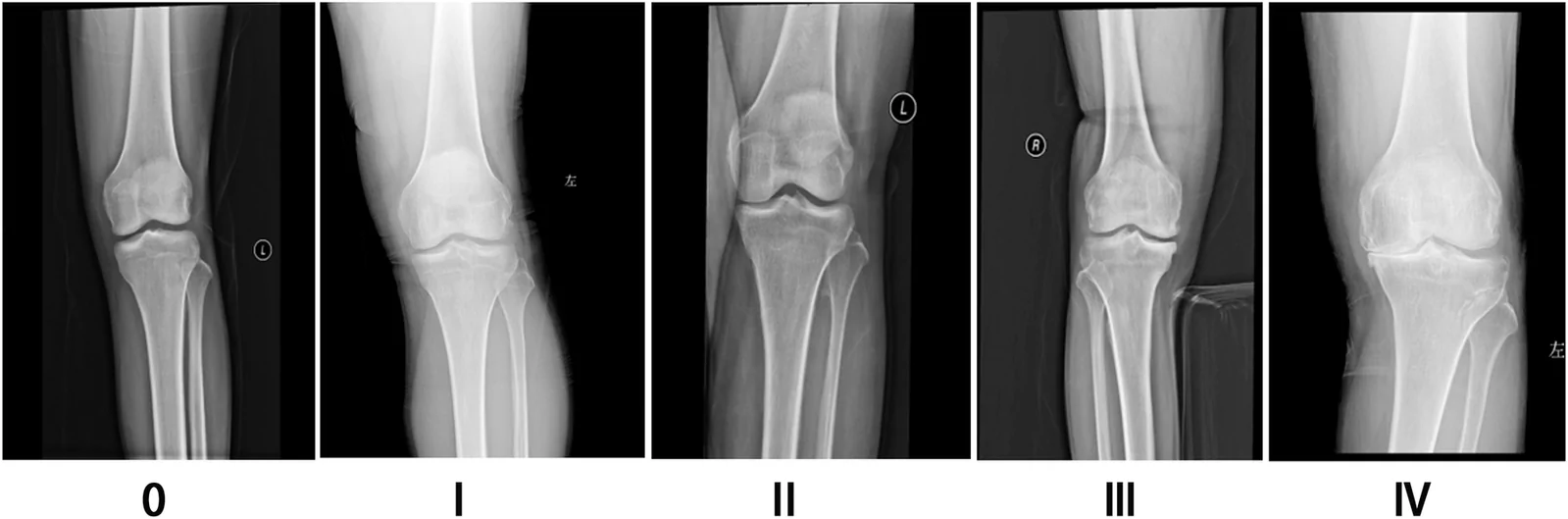
Radiographic staging of KOA structural severity utilizing the Kellgren-Lawrence (K-L) grading system.

## Weight intervention

From an epidemiological perspective, managing body composition represents the most impactful modifiable strategy for reducing the global incidence of KOA. Robust meta-analytic data indicate that customizable behavioral risk factors, predominantly overweight and obesity, account for approximately 14% of the overall risk driving radiographic KOA development (25). Crucially, the pathological trajectory of obesity-induced joint failure often initiates decades before clinical diagnosis; mid-life obesity serves as an aggressive predictor of subsequent KOA, and obese pediatric cohorts face a five-fold higher probability of maintaining an obese phenotype into adulthood (29). Consequently, mitigating this cumulative biomechanical and metabolic strain necessitates aggressive societal and public health frameworks implemented during early developmental stages (30). Strategically funded nutrition literacy, lifestyle modifications, and early-age physical activity programs are urgently required to arrest the metabolic syndrome pipeline, particularly in high-risk female populations, thereby shifting the therapeutic paradigm from reactive end-stage orthopedics to proactive lifelong prevention (31).

At the clinical intervention level, targeted weight reduction functions as a therapeutic cornerstone by simultaneously lowering mechanical focal shear stress on the knee joint and dampening systemic low-grade inflammation (32). Structured dietary and lifestyle modifications consistently induce symptomatic relief and functional restoration within weeks of initiation, exhibiting a clear dose-response relationship between the magnitude of adipose tissue loss and clinical benefit (33–35). International consensus guidelines universally mandate a multi-modal approach combining caloric restriction with tailored exercise regimens, establishing a minimum threshold of 5% to 10% weight loss to achieve clinically meaningful outcomes (32). The clinical superiority of this integration is highlighted by the landmark Intensive Diet and Exercise for Arthritis (IDEA) randomized controlled trial, which demonstrated that an 18-month combined diet-and-exercise protocol achieved a superior mean weight loss of 11.4%, drastically outperforming isolated dietary (9.5%) or exercise-only (2.0%) strategies (36, 37). Further elevating this therapeutic domain, contemporary evidence from the STEP 9 trial—which utilized weekly semaglutide therapy to achieve profound weight loss alongside dramatic functional optimization—underscores a revolutionary shift toward integrating metabolic pharmacotherapy within comprehensive joint preservation stratagems (38).

### Exercise therapy

As the non-pharmacological cornerstone of KOA management, exercise therapy optimizes the joint environment through intersecting biological and biomechanical pathways (2). At the cellular and tissue level, controlled mechanical loading cyclically drives synovial fluid dynamics, facilitating crucial nutrient perfusion and metabolic waste clearance within the avascular cartilage matrix, while concurrently dampening localized and systemic inflammatory signaling cascades to arrest chondrocyte apoptosis and subchondral bone resorption (39, 40). Concurrently, targeted training structurally modifies the knee biomechanical microenvironment by correcting periarticular muscular deficiencies, specifically quadriceps, hamstring, and hip abductor atrophy (41). By upgrading neuromuscular recruitment kinetics and dynamic shock-absorption, this structural reinforcement effectively unloads the vulnerable medial compartment and restores functional joint stability during weight-bearing ambulation (42). Exercise therapy represents the definitive, first-line non-pharmacological paradigm universally mandated by international consensus guidelines for the management of KOA.

#### Exercise modalities

Structured exercise therapy serves as the non-pharmacological cornerstone of knee osteoarthritis (KOA) management, shifting the intra-articular microenvironment from a catabolic cascade to an anabolic, homeostatic state (2, 43). To maximize joint preservation, traditional rehabilitation hinges on a strategic combination of land-based aerobic conditioning, progressive resistance strengthening, and aquatic therapy (43). Land-based aerobic modalities, predominantly tailored walking and stationary cycling, stimulate synovial fluid circulation to facilitate nutrient delivery and waste clearance within the avascular articular cartilage, while simultaneously downregulating key pro-inflammatory mediators including NF-*κ*B, IL-1β, and TNF-α (39, 40). To counteract periarticular muscular deficiencies, particularly quadriceps and hamstring atrophy, progressive strength training is mandatory (44). Mechanistically, establishing a minimum threshold increase of 30% to 40% in knee extensor strength via isometric, isotonic, or isokinetic muscle contractions is required to optimize neuromuscular recruitment, upgrade muscular shock-absorption, and effectively offload detrimental mechanical stresses from the tibiofemoral cartilage (41, 42). For patients restricted by severe mechanical pain, profound weakness, or elevated body mass index (BMI), aquatic therapy offers a critical low-impact alternative (45, 46). Utilizing the unique physical synergy of buoyancy and hydrostatic pressure, water-based resistance training systematically offloads axial joint stress, accelerates proprioceptive recovery, and promotes regional cartilage structural remodeling with significantly accelerated functional response rates compared to traditional terrestrial rehabilitation (47, 48).

Complementing these mechanical interventions, mind-body exercises—most notably Tai Chi and Yoga—have gained definitive clinical endorsement as core, universally accessible therapeutic modalities for long-term KOA management (49, 50). Rather than acting as mere general physical activities, these holistic regimens operate as highly efficient, multi-dimensional interventions that integrate physical postures with deep breath control and mental focus, yielding pain-relief profiles comparable to conventional aerobic strategies (51, 52). Biomechanically, the characteristic semi-squatting, lunging postures, and sequential asanas mandate precise, low-velocity controls over knee flexion and extension; this dynamic weight transfer substantially limits horizontal plane knee range of motion, maximizes quadriceps activation, and reduces the peak knee extension moment (53–55). Crucially, the therapeutic efficacy of mind-body training extends beyond localized joint mechanics into central nervous system pathways (56). Structured Tai Chi and Yoga regimens modulate the functional and structural connectivity of amygdala subregions, activating the parasympathetic nervous system to relieve protective muscle guarding, diminish pain-related fear, and suppress associated psychological distress such as anxiety and depression (56, 57). However, the clinical translation of these traditional mind-body modalities requires strict professional supervision and individualized movement modifications to ensure treatment fidelity, preserve a low knee adduction moment, and mitigate the risk of adverse musculoskeletal events (58).

#### Exercise strategies

The clinical efficacy of exercise therapy in KOA is strictly governed by a dose-response relationship structured around precise frequency and intensity thresholds (59, 60). Current clinical consensus establishes a frequency threshold of three sessions per week as the optimal paradigm for meaningful pain mitigation and functional restoration, routinely outperforming less frequent regimens (59, 61). Intriguingly, translational animal models suggest that when daily training volume is held constant, fractionating moderate loading into multiple shorter bouts per day yields superior chondroprotective and anti-inflammatory outcomes compared to a single continuous session, highlighting the temporal importance of joint offloading (62, 63). In terms of therapeutic intensity, while high-intensity protocols (such as aerobic training at 70%–80% heart rate reserve or resistance training at ≥80% 1RM) can enhance chondrocyte metabolism without sacrificing safety, network meta-analyses reveal comparable analgesic outcomes with low-intensity training, provided total caloric expenditure is matched (64, 65). For the substantial cohort of patients unable to tolerate high-load mechanical stresses, low-load training (20%–40% 1RM) coupled with blood-flow restriction (BFR) offers an equivalent functional surrogate (66). Mechanistically, optimizing this mechanical intensity is key to joint homeostasis; moderate-intensity loading directly drives chondrocyte autophagy and suppresses cellular pyroptosis via the P2X7/AMPK/mTOR signaling pathway, whereas light-intensity walking is sufficient to downregulate catabolic MMP-13 expression (67, 68).

Parallel to frequency and intensity, the temporal duration of exercise sessions dictates both peripheral joint kinetics and central neurochemical pathways. Synthesized clinical data validate a 60-minute structured session—comprising a 10-minute warm-up, 40-minute active training, and 10-minute cool-down—as the benchmark for maximizing functional gains (59). To prevent severe mobility loss and preserve baseline gait speed, a cumulative weekly threshold of at least 45 minutes of moderate-to-vigorous physical activity is clinically mandatory (69). This duration-dependent therapeutic benefit is heavily mediated by endogenous central analgesic mechanisms; sustained, long-duration exercise (70–90 minutes) upregulates the synthesis and systemic release of central endorphins and endogenous opioids far more efficiently than abbreviated sessions, effectively inducing central nervous system-mediated analgesia (70, 71). However, prolonged weight-bearing activities must be cross-referenced with radiographic disease severity, as extended unmonitored walking can increase joint contact forces and aggravate focal shear stress in advanced KOA, shifting the therapeutic goal from symptom reversal to mere functional arrest (72, 73). Ultimately, bridging the gap between theoretical activity guidelines and clinical practice mandates intensified, structured supervision coupled with targeted patient education, ensuring long-term patient compliance and optimization of the joint microenvironment (74, 75).

### Biomechanical therapy

Localized biomechanical aberrations, encompassing pathological obesity, periarticular muscular weakness, altered neuromuscular activation kinetics, and lower-limb malalignment, serve as primary catalysts driving abnormal joint contact forces and accelerating structural KOA progression. To mitigate these focal mechanical stressors, conservative management strategically integrates externally applied assistive devices and biomechanical therapies into multi-modal treatment frameworks, thereby synergistically amplifying the therapeutic ceiling of primary exercise interventions (76). Evidence from three-dimensional gait analysis underscores that suppressing the external knee adduction moment (KAM) during ambulatory cycles is directly coupled with robust clinical pain alleviation and optimized spatiotemporal functional outcomes. Within this framework, targeted innovations such as orthotic insoles, knee braces, and walkers function as pivotal structural modulators. Lateral wedge insoles are bio-mechanically engineered to counteract knee varus deformity—the structural hallmark of medial compartment KOA—by laterally shifting the ground reaction force vector, which consequently decompresses the vulnerable medial tibiofemoral interface (77). Similarly, specialized knee braces achieve profound symptomatic relief and functional optimization by modifying dynamic joint loading patterns, while unloader walkers drastically expand pain-free ambulatory capacity and enhance overall mobility metrics during weight-bearing activities (78, 79).

Despite the clear mechanical rationale supporting external orthoses, international clinical consensus mandates a nuanced, highly stratified prescription framework rather than a universal mandate. For instance, while short-term therapeutic advantages are frequently reported, the Osteoarthritis Research Society International (OARSI) guidelines caution against the indiscriminate, routine application of specific foot orthotics (80). This clinical divergence stems from a historically low certainty of pooled evidence, emphasizing that the therapeutic efficacy of wedged insoles is strictly contingent upon distinct individual biomechanical phenotypes, such as the severity of fixed structural varus alignment or concurrent foot and ankle abnormalities (80). Conversely, for patients presenting with profoundly compromised ambulatory stability or severe recalcitrant pain, the clinical deployment of unloader braces and gait-assistive equipment receives a strong diagnostic recommendation. This bifurcation in guidelines highlights that biomechanical interventions must transition away from a one-size-fits-all paradigm toward a highly personalized, phenotype-driven model of orthopedic rehabilitation (81).

### Physical therapy

Physical therapy encompasses a diverse spectrum of passive and semi-active modalities designed to optimize the intra-articular microenvironment, improve localized microcirculation, and alleviate protective muscle hypertonicity. However, these modalities differ substantially in their underlying biophysical mechanisms, clinical objectives, and the robustly established strength of clinical evidence (82).

As a core physical modality, Extracorporeal Shockwave Therapy (ESWT) supported by high-level clinical evidence plays a primary role in symptomatic KOA management. Biomechanically, ESWT delivers acoustic wave energy to periarticular soft tissues and subchondral bone, promoting microvascular circulation, suppressing localized neurogenic inflammation, and accelerating tissue repair cascades (83). Synthesized data from a systematic review and network meta-analysis of 32 randomized controlled trials (RCTs) demonstrate that ESWT provides substantial, durable pain reduction and functional improvement, firmly establishing it as a principal, high-evidence physical intervention for short-to-medium-term rehabilitation (84).

Serving an adjunctive role within conservative care, Manual Therapy (MT)—encompassing joint mobilization, passive manipulation, and soft-tissue stretching—focuses primarily on restoring passive joint range of motion and achieving temporary joint decompression (85). The therapeutic rationale hinges on reducing mechanical intra-articular pressure and suppressing catabolic NF-*κ*B inflammatory signaling through passive tissue distraction. While clinical trials confirm that MT delivers potent short-term analgesia superior to isolated resistance training during acute symptom exacerbations, its long-term clinical durability remains limited when applied as a monotherapy. Consequently, current clinical consensus positions MT strictly as a supportive, short-term adjunctive treatment designed to facilitate early participation in active exercise regimens rather than a standalone long-term intervention (82, 86).

In contrast, complementary approaches such as acupuncture offer adjunctive symptom relief but present an evidence-heterogeneous profile. While large-scale network meta-analyses show short-term pain and mobility gains, its mechanisms center on modulating synovial macrophage polarization and suppressing subchondral angiogenesis (87, 88). However, despite these plausible pathways, the clinical certainty of acupuncture remains limited by high trial heterogeneity, operator variability, and placebo effects (89). Consequently, it serves primarily as a secondary option for symptom modulation in tailored patient phenotypes rather than a core therapeutic intervention.

### Pharmacological management

The pharmacological management of KOA centers on systemic and topical NSAIDs and analgesics (90). While oral agents like etoricoxib and diclofenac effectively suppress prostaglandin-mediated pain and inflammation—with the former showing high potency and the latter a favorable safety profile (91), guidelines strongly favor topical NSAIDs as first-line interventions to bypass gastrointestinal toxicity (50, 92). Clinical consensus diverged regarding alternative pharmacotherapies, including acetaminophen, capsaicin, and duloxetine. Although acetaminophen is widely utilized for mild-to-moderate KOA pain owing to its gastrointestinal safety, its modest clinical efficacy and potential hepatotoxicity have triggered discordant recommendations between ACR/AF and OARSI guidelines (50, 80, 93). While duloxetine is conditionally integrated as an adjunctive option for centralized chronic pain, opioids have been systematically marginalized in current therapeutic pathways. Given their unfavorable safety profile and high risk of dependency, major clinical authorities, including AAOS and OARSI, strongly advocate for oral and topical NSAIDs as superior alternatives (50, 80).

For patients with refractory symptoms despite oral or topical pharmacotherapy, intra-articular (IA) injections of corticosteroids or hyaluronic acid (HA) offer targeted, localized alternatives, IA glucocorticoids rapidly suppress synovial inflammation, yielding moderate, short-term analgesia (typically under one month) and modest functional recovery (94). Consequently, they are endorsed by major bodies like ACR/AF and OARSI for acute flare-ups, though guidelines restrict their frequency (ideally to no more than once every four months) due to potential systemic absorption and accelerated joint degeneration, compounded by a lack of high-quality efficacy evidence beyond six weeks (94). In contrast, IA-HA aims to restore synovial fluid viscoelasticity, lubricating the joint, reducing shear forces, and stimulating chondrocyte metabolism to halt cartilage degradation (95). According to synthesized clinical evidence, HA administration yields moderate pain mitigation and functional improvements persisting for up to six months, especially in mild-to-moderate KOA patients unresponsive to initial conservative measures (96). While OARSI conditionally recommends HA for long-term management due to its superior safety and durability beyond 12 weeks (50), broader clinical consensus remains divided; ACR/AF positions HA as a secondary option for refractory cases, whereas AAOS opposes its routine administration citing questionable cost-effectiveness and heterogeneous clinical trial data (35, 95).

Formulated through the centrifugation of autologous whole blood, Platelet-Rich Plasma (PRP) represents a highly concentrated platelet fraction capable of secreting a complex cascade of bioactive cytokines and growth factors (97). Recent clinical evaluations continue to highlight both the therapeutic promise and the persistent debates surrounding its clinical efficacy. For instance, a meta-analysis pooling 12 randomized controlled trials (RCTs) demonstrated that leukocyte-poor PRP (LP-PRP) yielded significantly superior analgesic and functional outcomes compared to hyaluronic acid (HA) at 3, 6, and 12 months, exhibiting a comparable safety profile (98). Nevertheless, clinical consensus remains divided due to marked heterogeneity across study designs. Contrasting systematic reviews comparing PRP to saline placebos identified only modest, transient benefits that waned by 12 months, prompting researchers to question the true clinical utility of the therapy and emphasize the critical need for standardized PRP preparation protocols (99, 100).

Shifting away from conventional pharmacotherapy, contemporary KOA research focuses heavily on regenerative orthobiologics and targeted molecular inhibitors (101). Pro-inflammatory cytokines, most notably Interleukin-1 (IL-1) and Tumor Necrosis Factor (TNF), drive the catabolic cascades that accelerate cartilage destruction within the osteoarthritic joint (102–105). Clinical evaluations indicate that targeted IL-1 antagonism can successfully mitigate joint pain and restore physical mobility (101, 104). In parallel, TNF inhibitors such as etanercept have demonstrated therapeutic superiority over traditional sodium hyaluronate for early-stage pain control, underscoring the clinical promise of cytokine-directed interventions in halting progressive chondral degeneration (105, 106).

### Clinician supervision and education

Clinically, early-stage KOA patients and high-risk individuals frequently underestimate the necessity of timely intervention, largely due to their initially mild painful symptoms. Consequently, establishing long-term adherence to self-directed lifestyle modifications, such as home exercise and weight management, remains notoriously difficult. This highlights the critical role of structured clinician supervision and patient education, which require sustained oversight, regular follow-ups, and serial monitoring of clinical changes to halt KOA progression in its tracks. Drummen (107) demonstrated that clinically supervised exercise and patient education yield comparable, significant long-term improvements in pain, quality of life, and physical function for both early-stage and established KOA patients. Similarly, drawing from a massive cohort of over 32,000 participants, another longitudinal study highlighted that clinician-supervised exercise coupled with patient education constitutes a vital first-line strategy, driving equivalent and clinically meaningful long-term improvements for both knee and hip osteoarthritis patients (108). Furthermore, a recent 24-week randomized controlled trial revealed that for early-stage KOA, supervised exercise therapy provided superior pain relief and symptom mitigation compared to intra-articular platelet-rich plasma (PRP) injections (109). Given the robust efficacy of structured education and supervised physical programs, we advocate for the implementation of routine, supervised clinical follow-ups for high-risk cohorts and early-stage patients. Such oversight actively enforces therapeutic exercise and weight management—supplemented by targeted physical and pharmacological therapies when necessary—to prevent radiological progression, delay disease worsening, and ultimately reduce the long-term necessity for joint arthroplasty.

## Discussion

Knee osteoarthritis (KOA) represents a growing global public health challenge, yet contemporary research and clinical workflows remain disproportionately focused on late-stage surgical reconstruction, such as total knee arthroplasty (TKA), often neglecting the critical window for early secondary prevention and non-surgical management (12). To bridge this clinical gap, this review explicitly aims to establish a structured, “lifelong, stepped” non-surgical care paradigm specifically targeted at early-stage KOA and high-risk populations. By shifting the therapeutic paradigm from reactive end-stage orthopedics to proactive early interception, this study provides a clear directional focus on optimizing dynamic joint loading, dampening low-grade systemic inflammation, and preserving natural joint architecture before irreversible structural degradation occurs.

To realize this objective, we synthesized evidence across multi-modal conservative interventions and structured them into a practical, phenotype-targeted clinical pathway (Figure 2). As summarized in Table 2, foundational lifestyle modifications, specifically combined dietary weight loss and tailored physical exercise (aerobic, resistance, aquatic, and mind-body modalities like Tai Chi), serve as the strongly recommended core therapies to reduce focal mechanical stress and systemic inflammatory cascades. For individuals with persistent symptoms or specific structural deformities, therapeutic escalation incorporates phenotype-targeted adjuncts, including biomechanical orthoses (e.g., unloader braces), physical modalities (such as Extracorporeal Shockwave Therapy and manual therapy), and topical or systemic NSAIDs. In cases of refractory acute flares, intra-articular injections (corticosteroids, hyaluronic acid, or platelet-rich plasma) are utilized as temporary bridge therapies. Crucially, our synthesis highlights that continuous clinician supervision and structured patient education are essential to maintain long-term therapeutic adherence and prevent radiological disease progression (107).

**Figure 2:**
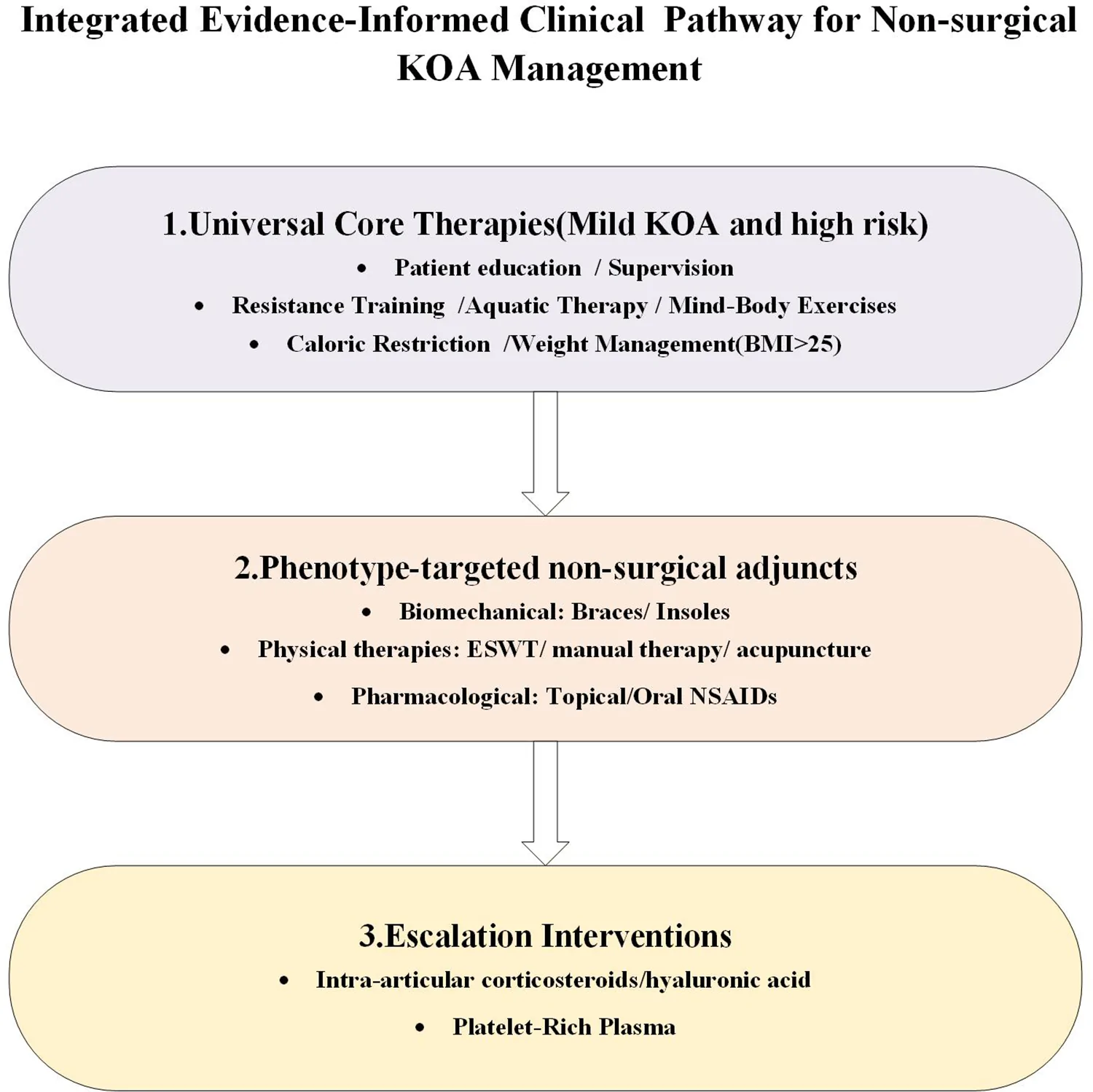
Visual synthesis of the integrated evidence-informed clinical pathway for non-surgical knee osteoarthritis management.

**Table 2:** Summary of evidence, clinical outcomes, and recommendations for Non-surgical knee osteoarthritis interventions.

Category	Intervention	Effects on pain	Physical function	Quality of life & secondary outcomes	Certainty of evidence (grade)	Clinical recommendation	Key references
Weight management	Dietary Caloric Restriction + Exercise	Significant reduction	Significant improvement	Reduces joint loads & inflammatory markers (IL-6, CRP); slows functional decline	High	Strongly Recommended	Messier et al., 2013; (37)
	Metabolic Pharmacotherapy (e.g., Semaglutide)	Substantial reduction	Substantial improvement	Substantial weight loss; improves overall cardiovascular profile	High	Conditional	Bliddal et al., 2024 (38)
Exercise therapy	Land-Based Aerobic & Resistance Training	Moderate-to-large reduction	Moderate-to-large improvement	Enhances muscle strength, dynamic stability, and gait kinetics	High	Strongly Recommended (Core Therapy)	Juhl et al., 2014; (61) Brophy et al., 2022 (35)
	Aquatic Therapy	Moderate reduction	Moderate improvement	Reduces axial compressive joint stress; high tolerance in severe pain/BMI	Moderate	Strongly Recommended	Ma et al., 2022; (45) Rewald et al., 2020 (46)
	Mind-Body Exercises (Tai Chi/Yoga)	Moderate reduction	Moderate improvement	Enhances proprioception, balance, and suppresses pain-related fear/anxiety	Moderate	Strongly Recommended	Wu et al., 2025; (51) Abafita et al., 2025 (55)
Biomechanical interventions	Unloader Knee Braces	Moderate reduction	Moderate improvement	Reduces external knee adduction moment (KAM) & dynamic joint loading	Moderate	Recommended (For specific alignment phenotypes)	Khosravi et al., 2021 (78)
	Lateral Wedge Insoles	Small/Inconsistent reduction	Minimal improvement	Variable KAM reduction; limited efficacy in fixed varus deformities	Low	Conditional/ Against Routine Use	Shaw et al., 2017; (77)
Physical therapies	Extracorporeal Shockwave Therapy	Moderate-to-large short/medium-term reduction	Moderate improvement	Promotes localized microvascular perfusion & dampens neurogenic inflammation	High	Conditional/ Supported	Lan et al., 2025 (84)
	Manual Therapy	Short-term reduction	Short-term improvement	Restores passive joint mobility; limited long-term durability as monotherapy	Low to Moderate	Conditional (Short-term Adjunct Only)	Runge et al., 2022 (86)
	Acupuncture	Moderate short-term reduction	Transient improvement	Suppresses synovial macrophage polarization;	Low	Conditional (Phenotype-Specific Adjunct)	Liu et al., 2024; (88)
Pharmacological management	Topical NSAIDs	Moderate reduction	Moderate improvement	Favorable safety profile; bypasses systemic/gastrointestinal adverse events	High	Strongly Recommended (First-line Analgesic)	Kolasinski et al., 2020; (80) Derry et al., 2017; (92)
	Oral NSAIDs	Moderate-to-large reduction	Moderate improvement	Effective for acute flares; carries gastrointestinal and cardiovascular risks	High	Conditional (Lowest dose, short duration)	da Costa et al., 2021 (91)
Intra-articular injections	Corticosteroids	Large short-term reduction (<4 weeks)	Transient improvement	Rapid flare control; risk of accelerated cartilage degradation with frequent repeat use	High (Short-term)/Very Low (Long-term)	Strongly Recommended (Acute flares only; max 3-4/year)	Juni et al., 2015
	Hyaluronic Acid	Small-to-moderate sustained reduction	Small-to-moderate improvement	Visco-supplementation & lubricin restoration; efficacy wanes beyond 6 months	Moderate	Conditional (Refractory mild-to-moderate KOA)	Bannuru et al., 2019; (50)
	PRP	Moderate reduction (up to 12 months)	Moderate improvement	Bioactive growth factor delivery; heterogeneous preparation & trial protocols	Low to Moderate	Conditional/Research Setting	Peng et al., 2025; (98)

Despite the clinical strengths and comprehensive nature of this stepped framework, several study limitations must be acknowledged. First, because this study was framed as a qualitative narrative review to synthesize a broad spectrum of heterogeneous non-surgical modalities, quantitative meta-analyses were not performed, which limits direct statistical head-to-head comparisons between distinct therapeutic classes. Second, while core interventions such as exercise and weight management are backed by high-certainty GRADE evidence, emerging modalities, including metabolic pharmacotherapy and orthobiologics, currently lack long-term, multi-center randomized controlled trial data regarding structural disease modification. Finally, the practical implementation of this stepped model relies heavily on long-term patient compliance and regional healthcare resources, which may vary across different clinical settings.

## Conclusion

As the global incidence and socioeconomic burden of knee osteoarthritis (KOA) continue to rise, clinical management remains disproportionately centered on late-stage surgeries, often overlooking the critical window for early prevention and conservative care. To bridge this gap, this narrative review outlines a proactive, stepped non-surgical framework designed to identify high-risk individuals early and intervene before joint degradation accelerates. Guided by clinician supervision and patient education, the strategy establishes weight control and tailored exercise as the therapeutic foundation. Treatment then escalates systematically to biomechanical adjustments, physical therapies, and targeted medications. Ultimately, this early, structured, and stepped approach offers a viable pathway to relieve pain, restore mobility, and delay or entirely avert the need for total joint replacement, substantially easing both individual suffering and the growing strain on global healthcare systems (Figure 3).

**Figure 3:**
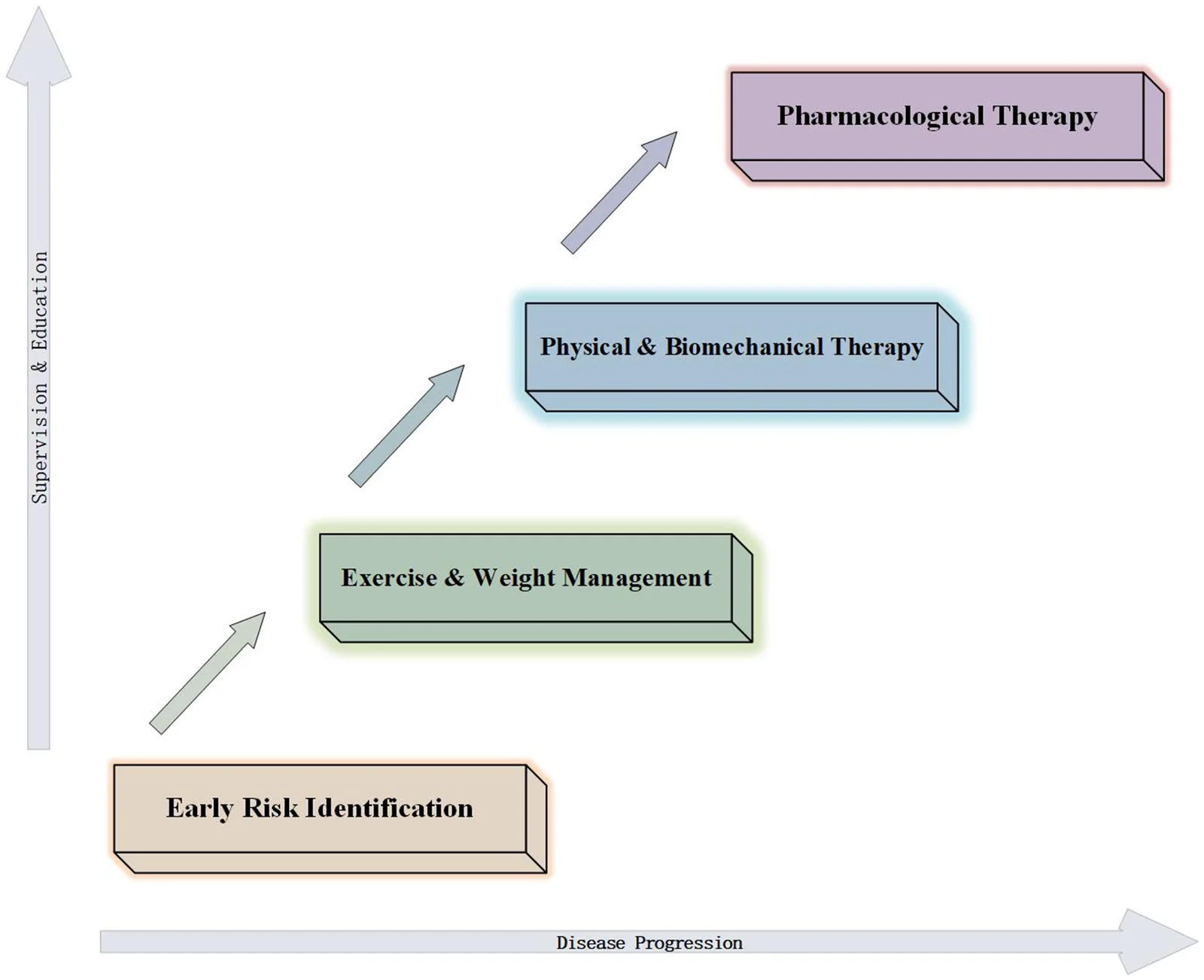
Schematic illustration of the stepped non-surgical intervention model for mild KOA or high-risk patients.
